# Reference ranges of myocardial T1 and T2 mapping in healthy Chinese adults: a multicenter 3T cardiovascular magnetic resonance study

**DOI:** 10.1186/s12968-023-00974-5

**Published:** 2023-11-16

**Authors:** Ziqian Xu, Weihao Li, Jiaqi Wang, Fei Wang, Bin Sun, Shifeng Xiang, Xiao Luo, Yanfeng Meng, Xiang Wang, Ximing Wang, Jianxun Song, Min Zhang, Dinghu Xu, Xiaoyue Zhou, Zhiguo Ju, Jiayu Sun, Yuchi Han, Yucheng Chen

**Affiliations:** 1 Department of Cardiology, West China Hospital, Sichuan University, Guoxue Xiang No. 37, Guo Xue Road, Chengdu, 610041 Sichuan People’s Republic of China; 2 Department of Radiology, West China Hospital, Sichuan University, Guoxue Xiang No. 37, Chengdu, 610041 Sichuan People’s Republic of China; 3 Department of Radiology, Anqing Municipal Hospital, Renmin Road No. 352, Yingjiang District, Anqing, 246003 Anhui People’s Republic of China; 4 Department of Radiology, Fujian Medical University Union Hospital, Xinquan Road No. 29, Fuzhou, 350001 Fujian People’s Republic of China; 5 Department of Radiology, Handan Central Hospital, Cong Taipei Road No. 59, Handan, 056002 Hebei People’s Republic of China; 6 Department of Radiology, Maanshan People’s Hospital, Hubei Road No. 45, Huashan District, Maanshan, 243099 Anhui People’s Republic of China; 7 Department of Radiology, Taiyuan Central Hospital, East Sandao Lane No. 5, Jiefang North Road, Xinghualing District, Taiyuan, 030009 Shanxi People’s Republic of China; 8 Department of Radiology, Wuhan Central Hospital, Shengli Street No. 26, Jiangan District, Wuhan, 430014 Hubei People’s Republic of China; 9 Department of Radiology, Shandong Provincial Hospital, Jingwuwei Seventh Road No. 324, Huaiyin District, Jinan, 250021 Shandong People’s Republic of China; 10 Department of Radiology, Shenzhen Baoan People’s Hospital, Longjing 2nd Road No. 118, Xinan Street, Baoan District, Shenzhen, 518101 Guangdong People’s Republic of China; 11 Department of Radiology, Beijing Hospital, Dongdan Dahua Road No. 1, Dongcheng District, Beijing, 100005 People’s Republic of China; 12 Department of Radiology, Nanjing Jiangning Hospital, Hushan Road No. 169, Jiangning District, Nanjing, 211199 Jiangsu People’s Republic of China; 13 MR Collaboration, Siemens Healthineers Ltd, Shanghai, People’s Republic of China; 14 College of Medical Imaging, Shanghai University of Medicine & Health Science, Shanghai, People’s Republic of China; 15 Cardiovascular Division, The Ohio State Wexner Medical Center, Columbus, OH USA

**Keywords:** Reference range, Phantom, T1 mapping, T2 mapping, Multicenter study

## Abstract

**Background:**

Although reference ranges of T1 and T2 mapping are well established for cardiovascular magnetic resonance (CMR) at 1.5T, data for 3T are still lacking. The objective of this study is to establish reference ranges of myocardial T1 and T2 based on a large multicenter cohort of healthy Chinese adults at 3T CMR.

**Methods:**

A total of 1015 healthy Chinese adults (515 men, age range: 19–87 years) from 11 medical centers who underwent CMR using 3T Siemens scanners were prospectively enrolled. T1 mapping was performed with a motion-corrected modified Look–Locker inversion recovery sequence using a 5(3)3 scheme. T2 mapping images were acquired using T2-prepared fast low-angle shot sequence. T1 and T2 relaxation times were quantified for each slice and each myocardial segment. The T1 mapping and extracellular volume standardization (T1MES) phantom was used for quality assurance at each center prior to subject scanning.

**Results:**

The phantom analysis showed strong consistency of spin echo, T1 mapping, and T2 mapping among centers. In the entire cohort, global T1 and T2 reference values were 1193 ± 34 ms and 36 ± 2.5 ms. Global T1 and T2 values were higher in females than in males (T1: 1211 ± 29 ms vs. 1176 ± 30 ms, p < 0.001; T2: 37 ± 2.3 ms vs. 35 ± 2.5 ms, p < 0.001). There were statistical differences in global T2 across age groups (p < 0.001), but not in global T1. Linear regression showed no correlation between age and global T1 or T2 values. In males, positive correlation was found between heart rate and global T1 (r = 0.479, p < 0.001).

**Conclusions:**

Using phantom-validated imaging sequences, we provide reference ranges for myocardial T1 and T2 values on 3T scanners in healthy Chinese adults, which can be applied across participating sites.

*Trial registration *URL: http://www.chictr.org.cn/index.aspx. Unique identifier: ChiCTR1900025518. Registration name: 3T magnetic resonance myocardial quantitative imaging standardization and reference value study: a multi-center clinical study.

**Supplementary Information:**

The online version contains supplementary material available at 10.1186/s12968-023-00974-5.

## Background

Cardiovascular magnetic resonance (CMR) mapping techniques allow non-invasive assessment of myocardial tissue characteristics and are increasingly routinely used in clinical studies. T1 and T2 mapping allows pixel-wise measurement of T1 and T2 relaxation times of left ventricular (LV) myocardium, as well as T1-derived extracellular volume (ECV) fraction, which can provide additional diagnostic and prognostic information [1]. T1 mapping has been utilized for the detection of myocardial fibrosis, edema, amyloid infiltration, iron overload, and lipid accumulation [1]. T2 mapping can be used to detect myocardial edema in acute ischemia and subclinical myocardial injury [2, 3], as well as myocardial inflammation involved in myocarditis [4, 5] and Takotsubo cardiomyopathy [6]. ECV is widely used in ischemic and nonischemic cardiomyopathies to quantify focal and diffuse fibrosis [7, 8].

Although both 1.5T and 3T scanners are widely used for cardiac imaging, reference ranges of T1 and T2 relaxation time values at 3T field strength have not been harmonized due to significant technical heterogeneity [9–11] and demographic-related discrepancies [12–16] (e.g., sex and age) between studies. The latest Society for Cardiovascular Magnetic Resonance (SCMR) guideline [17] emphasizes that local institutions should establish their own site-specific reference ranges for T1 and T2 mapping, and parameter values should only be compared under same technical conditions (e.g., vendors, field strength, acquisition scheme, and postprocessing approach). If local reference ranges are not available for T1 and T2 mapping, quantitative results should not be reported clinically [1]. The objective of this study is to provide reference ranges for myocardial T1 and T2 relaxation times derived from a large multicenter cohort and to verify the consistency among sites under controlled technical conditions.

## Materials and methods

### Study subjects

As part of a prospective clinical registry study, participants were recruited from 11 medical centers in China (URL: http://www.chictr.org.cn/index.aspx. Unique identifier: ChiCTR1900025518). From Sep 2019 to May 2022, a total of 1015 healthy Chinese adults without any known cardiovascular diseases (or other conditions affecting the cardiovascular system) and MRI contraindications were prospectively recruited. General clinical data were collected, including age, sex, height, weight, blood pressure, exercise intensity, personal and family history. Exclusion criteria were as follows: clinically confirmed hypertension; clinically confirmed diabetes; clinically diagnosed familial hyperlipidemia; myocarditis history; definite coronary artery disease; combined with structural heart disease, such as valvular disease (without mild valvular regurgitation), congenital heart disease; previous cardiac surgery; professional athletes or other long-term endurance sports, amateur marathon enthusiasts; clinically confirmed hyperthyroidism or hypothyroidism; combined with malignant tumors; combined with autoimmune diseases; severe lung disease; blood system diseases (including anemia); complicated with severe liver and kidney dysfunction; combined neurological and muscular diseases; drug abuse or addiction; recent (within 3 months) or long-term (more than 6 months) use of cardiovascular drugs; claustrophobia; no informed consent was obtained. Comprehensive physical examination and electrocardiogram were performed on the day of CMR scan.

### Ethics

The study was approved by the local Institutional Ethics Committee of each center, and all procedures were following the Declaration of Helsinki. All participants provided written informed consent.

### Acquisition and analyses of images

CMR was performed using 3T scanners (MAGNETOM Prisma or Skyra; Siemens Healthcare, Erlangen, Germany) equipped with a 32-channel phased-array body coil. For quality assurance of cardiac parametric mapping at each individual site, the T1 Mapping and Extracellular volume Standardization (T1MES) phantom scans were performed at each center prior to subject scanning. The T1MES phantom contained 9 vials (NiCl_2_ doped agarose) covering T1 and T2 ranges in the blood and myocardium before and after gadolinium-based contrast agents. Scanning was strictly performed based on the user manual (10.6084/m9.figshare.c.3610175_D1.v1). The temperature of the scanner room was 22 ± 2 ℃. The “Reference” T1 and T2 values (rT1, rT2) were first acquired using basic single-slice sequences with TR = 10 ms inversion recovery spin echo (SE, 8 Tis from 50 to 1800 ms) for measurement of T1, and single-slice TR = 7000 ms spin echo (SE) (25 TEs from 10 to 250 ms) for measurement of T2. The fitting of T1 relaxation time was performed using Grid Search-RD-NLS-PR algorithm [18]. T1 mapping was performed with a motion-corrected modified Look–Locker inversion recovery (MOLLI) sequence with a 5(3)3 scheme. The typical parameters of T1 mapping were as follows: true fast imaging with steady-state free precession pulse sequence; linear k-space ordering; repetition time (TR)/ echo time (TE) = 246 ms/1.08 ms; flip angle (FA) = 35°; inversion time (TI) = 100 ms; TI increment = 80 ms; field of view (FOV) = 150 × 150; matrix = 96 × 96; and slice thickness = 8 mm. The typical parameters for T2 mapping were as follows: T2-prepared fast low-angle shot (FLASH) sequence; centric k-space ordering; TR/TE = 228 ms/1.41 ms; FA = 12°; FOV = 150 × 150; matrix = 96 × 96; slice thickness = 8 mm; and T2 preparation pulses with 0-, 30-, and 55-ms echo times. RR intervals were set as 600 ms and 900 ms.

Cardiac DOT engine (Siemens Healthineers, Erlangen, Germany) was used for automatic localization of the long-axis planes and short-axis planes in all the scans to minimize the discrepancy between sites and technicians. T1 mapping was performed with a motion-corrected MOLLI sequence with a 5(3)3 scheme. T1 mapping slices were acquired at three long-axis planes (two-, three-, and four-chamber views) and three short-axis planes (the base, middle, and apex). The typical parameters were as follows: Trufi pulse sequence; linear k-space ordering; TR/ TE = 300 ms/1.15 ms; FA = 35°; TI = 100 ms; TI increment = 80 ms; FOV = 280 × 360; matrix = 256 × 160; and slice thickness = 8 mm. T2 mapping images were acquired using T2-prepared FLASH sequence in the same planes as T1 mapping. The typical parameters were as follows: centric k-space ordering; TR/TE = 315 ms/1.5 ms; FA = 12°; FOV = 280 × 360; matrix = 256 × 160; slice thickness = 8 mm; and T2 preparation pulses with 0-, 30-, and 55-ms echo times.

All images were analyzed in the core laboratory with dedicated postprocessing software (Medis suite v2.3; Medis Medical Imaging, Leiden, the Netherlands). Image analysis was performed by two investigators (Z.Q.X and W.H.L, with 9 and 7 years of CMR experience, respectively). T1 and T2 values were measured for each of the 9 vials in identically sized ROIs that occupied the central 50% area of each gel, away from edge-ringing artifacts. T1 and T2 mapping of subjects were analyzed according to the SCMR post-processing guideline [17]. When measuring T1 and T2 values, the endo- and epicardial contours were manually delineated with 10% endo- and epicardial offsets. T1 and T2 mapping were assessed per segment according to the American Heart Association (AHA) 16-segment model. Segments with artefacts, basal images with outflow tract, and incomplete apical images were excluded from analysis.

### Intra-observer and inter-observer variability

To test the intra-observer and inter-observer variability in T1 and T2 assessment, 50 subjects were randomly selected. Inter-observer variability was analyzed on the same image set by two independent investigators. Intra-observer variability was analyzed on the same image set by one investigator one month later.

### Statistical analysis

Statistical analysis was performed using SPSS (version 26.0, IBM SPSS Inc., Chicago, IL, USA) and R (version 4.0.3; the Free Software Foundation’s GNU project). Continuous variables were tested for normality using the Kolmogorov–Smirnov test. The reference ranges were defined as ± 2 standard deviation (SD) from the mean. Kendall’s W was performed to verify the consistency of SE, T1 mapping, and T2 mapping among centers. The coefficient of variation (CoV) was defined as the ratio of the SD to the mean. Accuracy was measured in each vial as average of absolute difference between T1 (T2) measurements obtained from SE and each sequence. Precision was measured in each vial as the average of the SD of T1 (T2) measurements obtained with each sequence. T1 and T2 values were compared among age groups (19–39 years, 40–59 years and > 60 years) and between sexes. Independent-samples t-tests were used to compare mean values between sexes. One-way analysis of variance or Kruskal–Wallis test were used to compare mean values among three age groups. Linear regression was used for analyses of the relationships between global T1, T2 and age. Intra-observer and inter-observer variability were measured by the CoV, intraclass correlation coefficient (ICC), and Bland–Altman analysis. A two-tailed p value of < 0.05 was considered statistically significant. All the subjects were also stratified by sex and age decades, shown in Additional file 1: Table S1 and S2.

## Results

### Phantom study

**Fig. 1:**
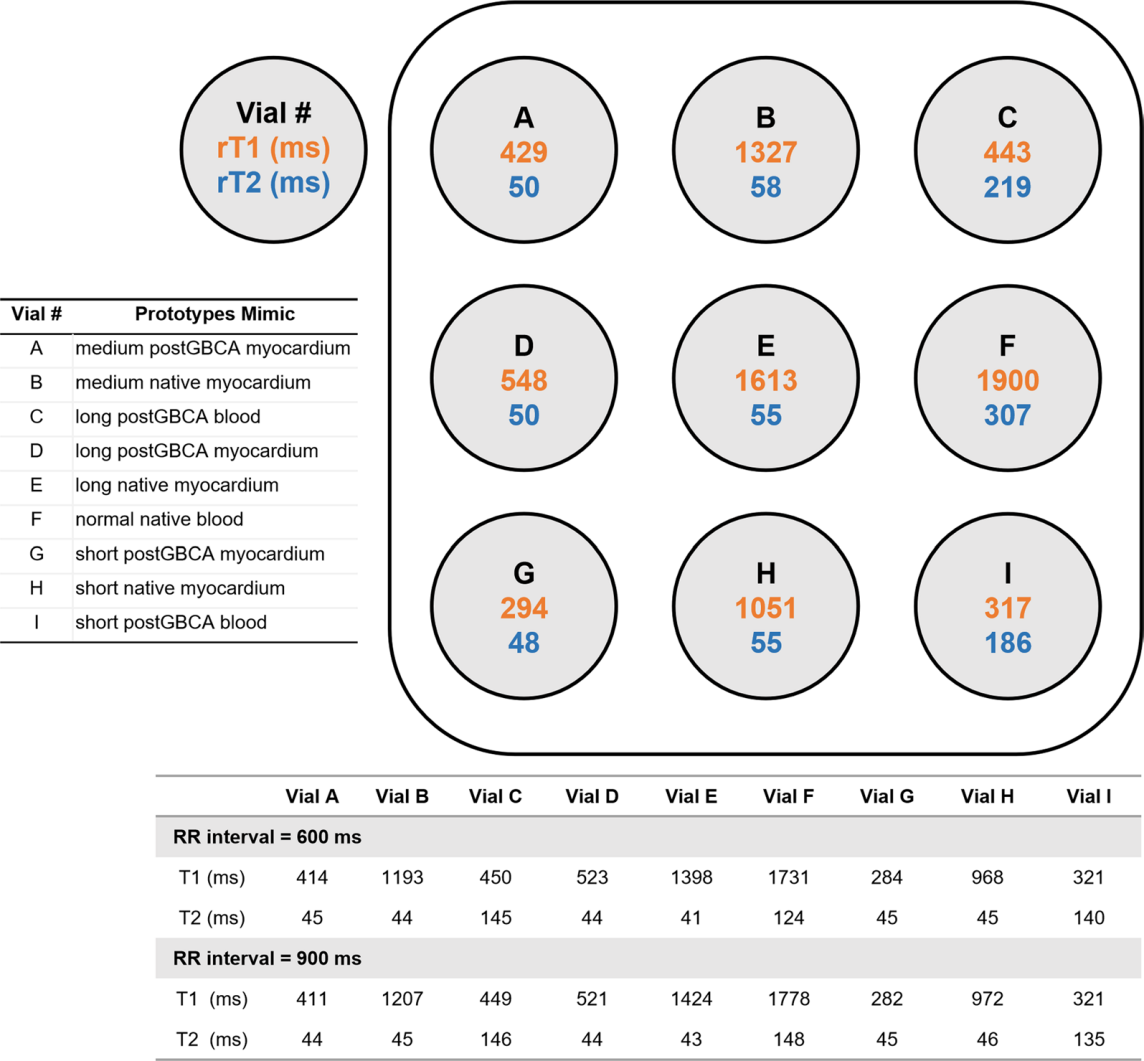
Phantom compartment arrangement and T1 and T2 values

The mean rT1, rT2, T1 and T2 values for each vial are shown in Fig. 1. The consistency of rT1 and rT2 among centers was almost perfect (Kendall’s W = 0.992; 0.988, respectively), indicating that the SE sequences are reliable. The agreement of T1 mapping among centers was almost perfect (Kendall’s W = 0.992, RR interval = 600 ms; Kendall’s W = 0.992, RR interval = 900 ms), and the agreement of T2 mapping among centers was strong (Kendall’s W = 0.825, RR interval = 600 ms; Kendall’s W = 0.885, RR interval = 900 ms), which indicates the reliability of T1 mapping and T2 mapping data among centers, even at a fast heart rate. In native myocardium mimics (vial B, E, and H), the CoVs for T1 mapping and T2 mapping were below 10% (Table 1), indicating low variability. The accuracy and precision of T1 mapping and T2 mapping were shown in Table 1.

**Table 1:** The CoV, accuracy, and precision of T1 mapping and T2 mapping of T1MES phantom

Vial no.	T1 mapping			T2 mapping		
	CoV (%)	Accuracy (ms)	Precision (ms)	CoV (%)	Accuracy (ms)	Precision (ms)
*RR interval = 600 ms*						
Vial A	0.9	15	0.9	12.1	5	11.0
Vial B	3.6	134	3.2	6.3	14	4.7
Vial C	0.5	6	0.5	2.3	74	1.5
Vial D	0.8	25	0.7	4.8	6	4.2
Vial E	5.0	215	4.3	6.6	14	4.9
Vial F	7.9	169	7.2	17.2	183	6.9
Vial G	1.0	10	0.9	2.8	2	2.7
Vial H	2.7	82	2.5	6.1	10	5.0
Vial I	19.8	5	20.1	12.2	46	9.2
*RR interval = 900 ms*						
Vial A	0.5	17	0.4	4.0	6	3.5
Vial B	3.6	119	3.3	4.3	13	3.4
Vial C	0.8	6	0.8	2.2	72	1.4
Vial D	0.6	27	0.6	4.1	6	3.7
Vial E	4.4	189	3.8	5.2	12	4.0
Vial F	7.1	122	6.7	10.0	159	4.8
Vial G	0.7	11	0.7	2.3	2	2.2
Vial H	2.2	79	2.1	3.9	9	3.3
Vial I	19.9	4	20.1	8.9	51	6.5

The coefficient of variation (CoV) was defined as the ratio of the SD to the mean. Accuracy was defined as average of absolute difference between T1 (T2) measurements obtained from T1 (T2) SE and T1 (T2) mapping. Precision was defined as the average of the SD of T1 (T2) measurements obtained with T1 (T2) mapping

### Demographic characteristics of healthy Chinese subjects

A total of 1015 subjects (515 men, 42 ± 15 years) were included, the demographic characteristics of study subjects are shown in Table 2. There was no significant difference in age between men and women (p = 0.240). Body mass index (BMI), systolic blood pressure (SBP), diastolic blood pressure (DBP) and heart rate (HR) were higher in males compared to females. Biventricular end-diastolic/systolic volume index, and LV mass index were higher in males than in females, while LVEF and RVEF were higher in females than males.

**Table 2:** Demographic characteristics of multicenter cohort of healthy Chinese adults

	Total (n = 1015)	Male (n = 515)	Female (n = 500)	P value
Age (years)	42 ± 15	42 ± 15	43 ± 14	0.240
BMI (kg/m^2^)	23.3 ± 3.2	24.3 ± 3.1	22.3 ± 3.0	< 0.001
SBP (mmHg)	121 ± 11	122 ± 110	118 ± 11	0.049
DBP (mmHg)	74 ± 9	75 ± 9	71 ± 9	< 0.001
h (bmp)	75 ± 11	76 ± 12	75 ± 10	0.027
LVEDVi (ml/m^2^)	75.2 ± 11.4	77.7 ± 12.0	72.7 ± 10.2	< 0.001
LVESVi (ml/m^2^)	28.4 ± 5.9	30.0 ± 6.1	26.8 ± 5.2	< 0.001
LVEF (%)	62.3 ± 4.7	61.4 ± 4.7	63.2 ± 4.6	< 0.001
LVMi (g/m^2^)	45.5 ± 7.3	49.1 ± 7.0	41.8 ± 5.5	< 0.001
RVEDVi (ml/m^2^)	75.4 ± 13.1	79.5 ± 13.9	71.2 ± 10.9	< 0.001
RVESVi (ml/m^2^)	28.8 ± 6.5	31.3 ± 6.5	26.3 ± 5.4	< 0.001
RVEF (%)	62.0 ± 4.7	60.9 ± 4.5	63.2 ± 4.6	< 0.001

Data are presented as means ± standard deviation (SD). P-value is for t-test between sexes

*BMI* body mass index, *SBP* systolic blood pressure, *DBP* diastolic blood pressure, *HR* heart rate, *LV* left ventricle/ventricular, *RV* right ventricle/ventricular, *EDVi* end-diastolic volume index, *ESVi* end-systolic volume index, *EF* ejection fraction, *LVMi* left ventricular mass index

### Sex and age differences in myocardial T1 and T2 values

A total of 681 (4.2%) myocardial segments for T1 mapping and 912 (5.6%) myocardial segments for T2 mapping were excluded. The reference ranges for T1 and T2 are shown in Table 3. Both T1 and T2 were significantly higher in females when compared to males (both p < 0.001). The values are shown with the subjects divided into three groups according to age range (19–39 years, 40–59 years and ≥ 60 years). Global and septal T2 progressively increased with age (both p < 0.001), while there was no significant difference in global and septal T1 among the three age groups (Table 4).

**Table 3:** T1 and T2 mapping parameters in healthy Chinese adults by sex

	Total (n = 1015)	Male (n = 515)	Female (n = 500)	P value
*T1 value (ms)*				
Base	1195.5 ± 35.8 (1123.9–1267.1)	1180.3 ± 32.4 (1115.5–1245.2)	1211.1 ± 32.2 (1146.7–1275.6)	< 0.001
Middle	1186.5 ± 39.0 (1108.5–1264.5)	1169.4 ± 34.6 (1100.2–1238.6)	1204.1 ± 35.3 (1133.5–1274.6)	< 0.001
Apex	1201.5 ± 46.2 (1109.1–1293.8)	1180.0 ± 42.2 (1095.6–1264.3)	1225.0 ± 38.2 (1148.5–1301.4)	< 0.001
Global	1193.2 ± 34.1 (1124.9–1261.5)	1176.0 ± 29.9 (1116.3–1235.7)	1210.9 ± 28.8 (1153.2–1268.6)	< 0.001
Septum	1201.9 ± 42.2 (1117.6–1286.3)	1185.5 ± 39.5 (1106.4–1264.5)	1218.9 ± 37.9 (1143.2–1294.6)	< 0.001
*T2 value (ms)*				
Base	35.7 ± 2.6 (30.5–40.9)	35.0 ± 2.5 (30.0–40.0)	36.4 ± 2.5 (31.4–41.4)	< 0.001
Middle	35.9 ± 2.7 (30.4–41.3)	35.1 ± 2.7 (29.7–40.5)	36.7 ± 2.5 (31.6–41.7)	< 0.001
Apex	36.5 ± 3.1 (30.4–42.6)	35.8 ± 3.1 (29.7–41.9)	37.2 ± 2.9 (31.4–43.0)	< 0.001
Global	35.9 ± 2.5 (30.9–41.0)	35.2 ± 2.5 (30.2–40.2)	36.7 ± 2.3 (32.0–41.4)	< 0.001
Septum	36.3 ± 3.0 (30.3–42.3)	35.6 ± 3.0 (29.6–41.6)	37.0 ± 2.8 (31.5–42.6)	< 0.001

Data are presented as means ± SD (lower/upper limits). Lower/upper limits calculated as mean ± 2 SD. P-value is for t-test between sexes

**Table 4:** Parameters of T1 and T2 mapping in healthy Chinese adults by age

	19–39 years (n = 467)	40–59 years (n = 425)	≥ 60 years (n = 123)	P value
*T1 value (ms)*				
Base	1192.4 ± 32.7 (1127.1–1257.8)	1196.2 ± 38.2 (1119.7–1272.6)	1205.1 ± 37.1 (1131.0–1279.2)	0.966
Middle	1186.1 ± 37.9 (1110.4–1261.8)	1185.4 ± 39.1 (1107.2–1263.7)	1191.4 ± 42.7 (1106.1–1276.7)	0.030
Apex	1204.2 ± 42.9 (1118.4–1290.0)	1198.6 ± 48.4 (1101.8–1295.4)	1200.8 ± 50.0 (1100.7–1300.9)	0.262
Global	1192.6 ± 32.1 (1128.4–1256.7)	1192.3 ± 35.4 (1121.5–1263.0)	1198.7 ± 37.0 (1124.6–1272.7)	0.178
Septum	1201.1 ± 40.1 (1120.8–1281.4)	1200.0 ± 42.2 (1115.6–1284.4)	1211.6 ± 48.1 (1115.4–1307.9)	0.156
*T2 value (ms)*				
Base	35.1 ± 2.7 (29.8–40.5)	36.0 ± 2.4 (31.2–40.8)	36.8 ± 2.5 (31.8–41.7)	< 0.001
Middle	35.3 ± 2.8 (29.7–40.9)	36.2 ± 2.5 (31.1–41.2)	37.0 ± 2.7 (31.6–42.4)	< 0.001
Apex	35.9 ± 3.1 (29.6–42.2)	36.9 ± 2.9 (31.0–42.7)	37.5 ± 2.8 (31.9–43.1)	0.099
Global	35.4 ± 2.6 (30.2–40.6)	36.2 ± 2.3 (31.6–40.9)	37.0 ± 2.4 (32.2–41.8)	< 0.001
Septum	35.8 ± 3.1 (29.6–42.0)	36.5 ± 2.8 (30.9–42.2)	37.3 ± 2.8 (31.6–43.0)	< 0.001

Data are presented as means ± SD (lower/upper limits). Lower/upper limits calculated as mean ± 2 SD. P-value is for one-way ANOVA test among age groups

### Analysis of relationships

For both sexes, linear regression showed that no correlation between age, BMI, and global T1 or T2. In males, a positive correlation was found between HR and global T1 (r = 0.479, p < 0.001), but not for global T2. In females, no correlation was found between HR and global T1 or global T2.

### Segmental and layer-specific analysis of myocardial T1 and T2 values

**Fig. 2:**
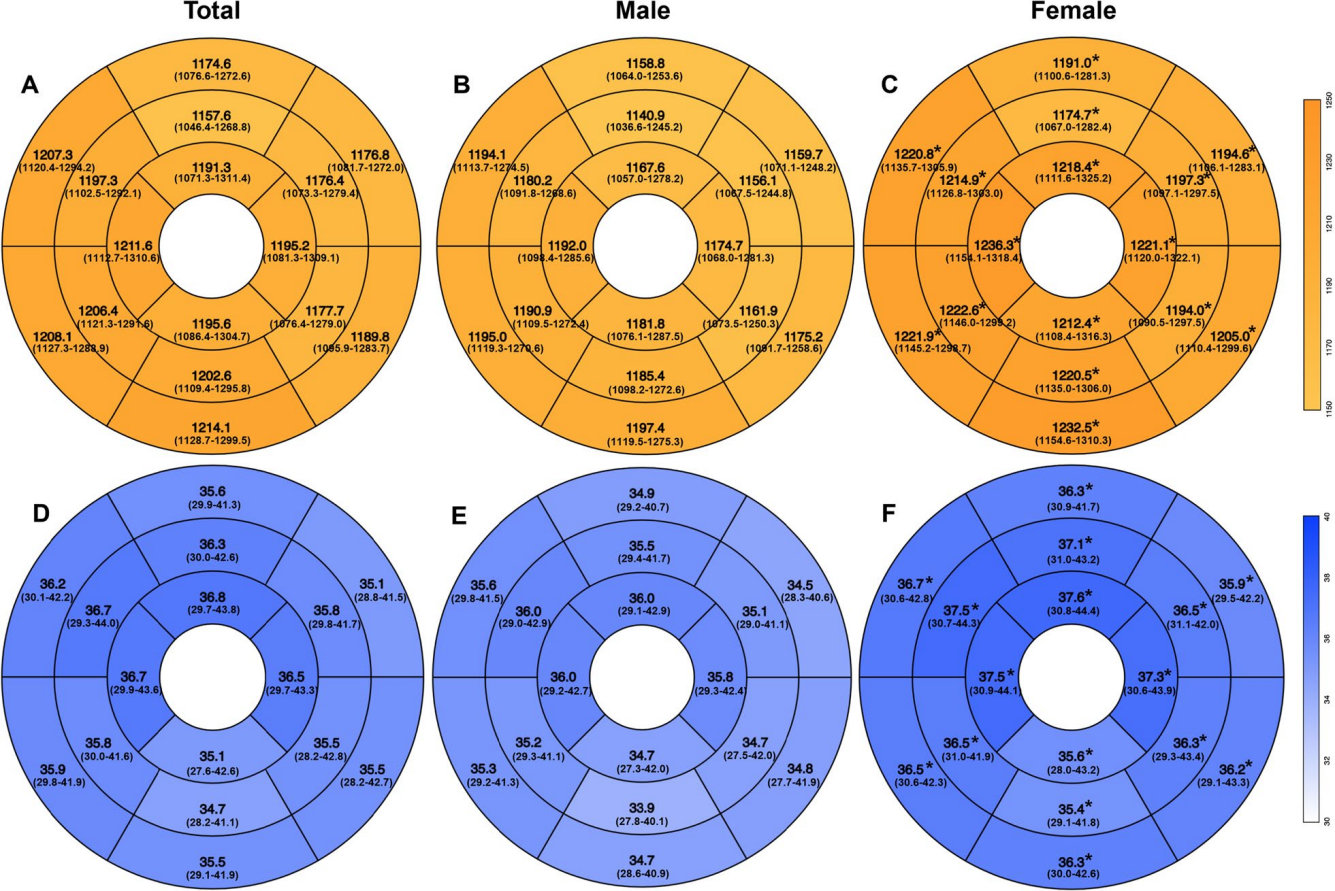
T1 and T2 value in AHA-16 segments displayed in bulls eye plots. T1 value (**A–C**) and T2 value (**D–F**) in AHA-16 segments. *p < 0.001, male vs. female

Segmental T1 and T2 values according to the AHA 16-segment model are shown in the bulls-eye plots in Fig. 2). T1 and T2 values of all segments in females were higher than those in males. For the entire cohort, T1 and T2 values were statistically different among the three slices (all p < 0.001), and apical T1 and T2 values were higher compared to basal (both p < 0.01) and middle slice values (both p < 0.01). In males, T1 and T2 values were also statistically different among the three slices (both p < 0.001), and the apical T2 values were higher than the basal (both p < 0.001) and middle slices (both p < 0.001) but not T1. In females, T1 and T2 values were statistically different among the three slices (all p < 0.001), and the apical T1 and T2 values were higher than the basal (both p < 0.01) and middle slices (both p < 0.01).

### Intra-observer and inter-observer reproducibility

**Fig. 3:**
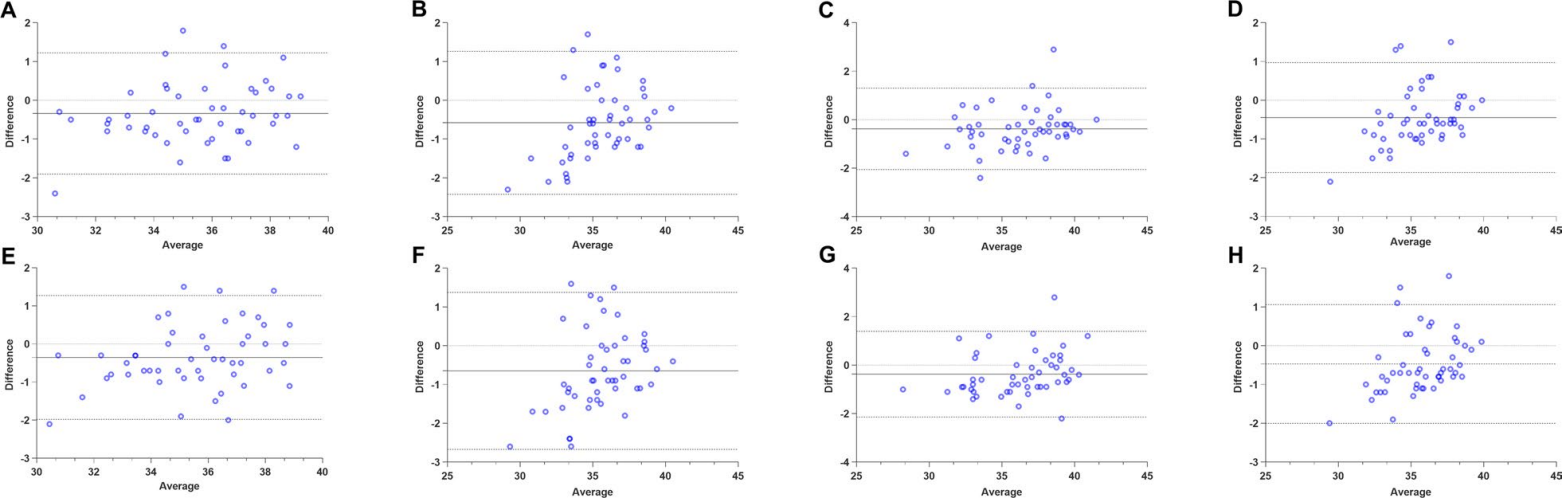
Bland–Altman plots for intra-observer and inter-observer reproducibility of T1 mapping. Bias (solid lines) and 95% limits of agreement (dashed lines) are shown. The first row shows the intra-observer reproducibility of T1 base (**A**), T1 middle (**B**), T1 apex (**C**), and T1 global (**D**). The second row shows the inter-observer reproducibility of T1 base (**E**), T1 middle (**F**), T1 apex (**G**), and T1 global (**H**)

**Fig. 4:**
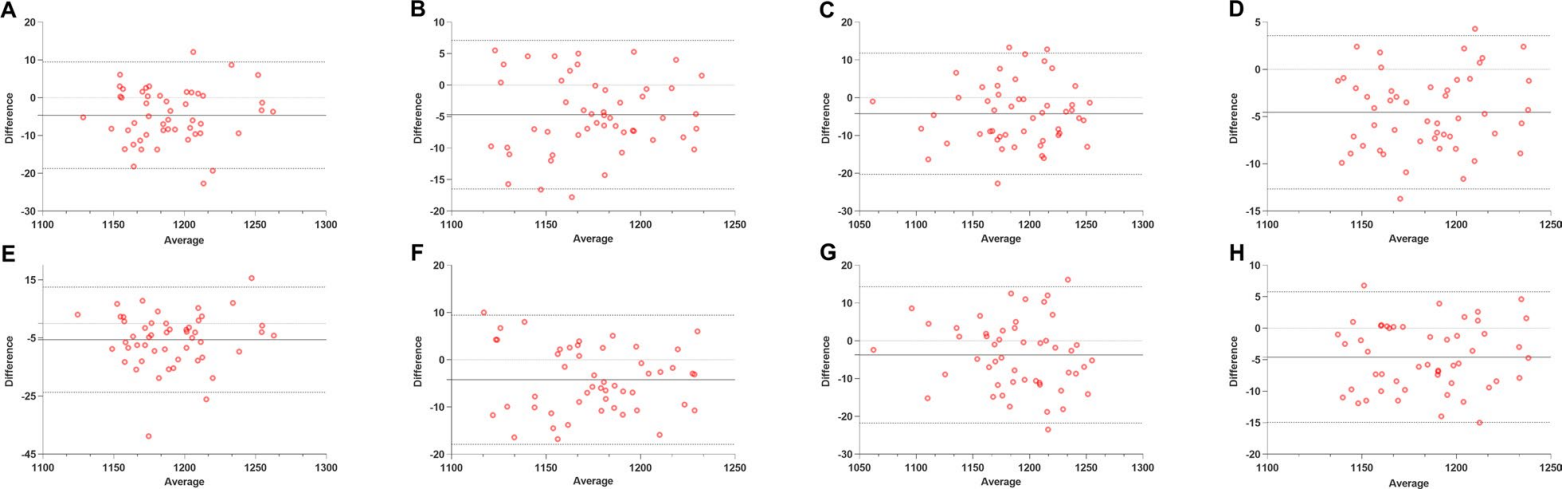
Bland–Altman plots for intra-observer and inter-observer reproducibility of T2 mapping. Bias (solid lines) and 95% limits of agreement (dashed lines) are shown. The first row shows the intra-observer reproducibility of T2 base (**A**), T2 middle (**B**), T2 apex (**C**), and T2 global (**D**). The second row shows the inter-observer reproducibility of T2 base (**E**), T2 middle (**F**), T2 apex (**G**), and T2 global (**H**)

The intra-observer and inter-observer variabilities of the T1 and T2 values are shown in Table 5; Figs. 3 and 4. T1 and T2 exhibited high ICCs and low CoV values, indicating excellent reproducibility.

**Table 5:** Intra-observer and inter-observer reproducibility of T1 and T2 values

	Bias	SD of bias	95% LOA	CoV (%)	ICC	p value
*Intra-observer*						
T1 base	4.63	7.19	(− 18.73 to 9.46)	2.6	0.962	< 0.001
T1 middle	− 5.19	6.14	(− 17.23 to 6.85)	2.6	0.970	< 0.001
T1 apex	− 4.23	8.19	(− 20.28 to 11.83)	3.5	0.976	< 0.001
T1 global	− 4.54	4.14	(− 12.65 to 3.57)	2.4	0.978	< 0.001
T2 base	− 0.34	0.80	(− 1.90 to 1.20)	6.2	0.926	< 0.001
T2 middle	− 0.58	0.94	(− 2.42 to 1.27)	6.6	0.892	< 0.001
T2 apex	− 0.38	0.86	(− 2.06 to 1.30)	7.9	0.948	< 0.001
T2 global	− 0.45	0.72	(− 1.87 to 0.97)	6.3	0.930	< 0.001
*Inter-observer*						
T1 base	− 5.58	9.23	(− 23.68 to 12.52)	2.6	0.939	< 0.001
T1 middle	− 4.92	7.62	(− 19.84 to 10.01)	2.7	0.967	< 0.001
T1 apex	− 3.72	9.20	(− 21.75 to 14.32)	3.6	0.973	< 0.001
T1 global	− 4.58	5.29	(− 14.95 to 5.78)	2.5	0.972	< 0.001
T2 base	− 0.35	0.83	(− 1.98 to 1.28)	6.1	0.917	< 0.001
T2 middle	− 0.65	1.03	(− 2.67 to 1.38)	6.6	0.869	< 0.001
T2 apex	− 0.37	0.90	(− 2.14 to 1.40)	7.9	0.943	< 0.001
T2 global	− 0.47	0.78	(− 2.00 to 1.06)	6.2	0.918	< 0.001

*LOA*  limit of agreement, *CoV* coefficient of variation, *ICC* intra-class correlation coefficient, *SD* standard deviation

## Discussion

In this large multicenter 3T CMR study, using a standardized scanning platform and protocol, as well unified post-processing in a core laboratory, reference ranges for myocardial T1 and T2 relaxation times were established. The reference ranges show discrepancies with previous 3T single center studies. Although no significant correlations were found between age, sex, and myocardial T1 or T2 values, sex and age differences are confirmed. In addition, phantom analysis demonstrates the transferability of the T1 and T2 mapping protocols beyond a single site, which provides a viable basis for multicenter CMR studies in heart disease utilizing mapping techniques.

Currently, the most widely used clinical techniques for T1 mapping are based on the MOLLI or shortened-MOLLI (shMOLLI) techniques [19]. Myocardial T1 reference values reported in previous studies differed significantly due to different field strength, vendors, and pulse sequences. Gottbrecht et al. [10] have summarized the pooled mean of native T1 at 3T ranging from 1171 to 1214 ms (Siemens, MOLLI) and 1152–1188 ms (Siemens, shMOLLI). Although the MOLLI 5(3)3 scheme with a total acquisition duration of 11 heartbeats has excellent precision and reduced HR sensitivity [20–22], there is still no consensus on the T1 reference value, which partly limits their use in clinical routine.

Previous studies have reported conflicting information on age or sex associations of myocardial T1 values [12–15, 23, 24]. T1 was found to be associated with older age in men in the Multi-Ethnic study of Atherosclerosis (MESA) (n = 1231, age range: 54–93 years) [12], while Rauhalami et al. [24] demonstrated that global T1 was inversely associated with age in women (n = 84, age range: 45 ± 18 years). Dong et al. [15] found that sex was independently related with global T1 (n = 69, age range: 18–76 years), while Dabir et al. [23] found no correlation between age, sex, and T1 (n = 102, age range: 17–83 years). Some studies have interpreted the age-related elevation of myocardial T1 as a marker of increased interstitial fibrosis or ECV [12, 15]. Conversely, Rosmini et al. [25] demonstrated myocardial T1 measured by MOLLI and shMOLLI was slightly lower with increasing age (n = 94, age range: 20–76 years), which may be attributed to the accumulation of myocardial lipofuscin or hemosiderin with age. We found no relationship between age and myocardial T1 in this study. The discrepancies between the findings suggest that the characterization of healthy aging myocardium is challenging, with limited data available in the field. Possible reasons for this finding are as follows: First our subjects were free of cardiovascular disease and associated comorbidities, the prevalence of which increases with age and affects myocardial T1. Second, our subjects included populations from different provinces, and the interference of geographic factors was weakened. Third, native T1 may be insensitive to detect mild fibrosis in healthy aging myocardium.

Myocardial T2 relaxation curves are constructed based on the multi-echo pulse sequences, and the most frequently used techniques are T2p-bSSFP and gradient spin echo (GRASE) supplied by different vendors. Myocardial T2 reference values reported in previous studies were significantly different, ranging from 32 to 47 ms (Siemens, T2p-bSSFP) [26–29] at 3T. Additionally, a meta-analysis demonstrated the pooled mean of T2 was 44–48 ms at 3T [11]. The significant heterogeneity of T2 values among studies suggest that T2 is sensitive to technical factors, e.g., vendor differences, field strength, pulse sequence, echo times, off-resonance, motion compensation, fit model, and B1 inhomogeneity [30, 31], emphasizing the need to establish site-specific reference for specific technical conditions. In addition, sex and age differences among study cohorts may also have additional effects on T2 heterogeneity. However, due to the small sample size of myocardial T2 values reported in previous studies, most of which number fewer than 50 individuals, the relevant effects of demographic factors have not been well established. Bönner et al. [32] demonstrated higher myocardial T2 in females and age-related increase T2 measured by 1.5T GRASE, while Roy et al. [14] found myocardial T2 measured by 3T GRASE decreased with increasing age by but are not affected by sex. Although we found no relationship between myocardial T2 and age, there is a statistically significant difference in T2 value of each age group. Establishing the reference ranges at 3T based on a large cohort of healthy individuals and standardized techniques will encourage more widespread application of T2 mapping techniques.

We observed slightly higher T1 and T2 values in the apex than in the basal and mid myocardium. Partial volume effect due to the LV curvature could explain this finding by including the blood signal into voxels [33]. We attempted to minimize this effect by choosing a high isotropic spatial resolution and excluding part of endocardium. We also observed slight difference in T1 and T2 values per segment. This phenomenon may be attributed to artifacts (e.g., B0, B1 inhomogeneity, off-resonance artifacts, motion artifacts, susceptibility artifacts, and partial volume) [33, 34] and physiologic variance (e.g., regional difference in perfusion, ECV, amount of collagen, and collagen fiber orientation) [9]. A small number of segments in this study were excluded from analysis due to motion artifacts and susceptibility artifacts. The main cause of artifacts at 3T is B0 and B1 inhomogeneity resulting in segmental differences. In addition, partial volume effects in thicker slices can lead to artifacts in the hypermobile regions, such as the left ventricular lateral wall [9, 33]. Excluding segments affected by artifacts did not result in excluding extreme outliers in a few previous studies [9, 34]. The intra-individual inhomogeneity of myocardial segments deserves further investigation. Although the slice and segmental differences were small, this must be taken into consideration in clinical interpretation because of possible overlap between healthy and abnormal tissue. In addition, some cardiomyopathies often show segmental involvement, for example, myocardial fibrosis and edema in hypertrophic cardiomyopathy often appear in the most hypertrophic segment [35, 36], and Fabry disease typically shows fibrosis and edema in the basal and mid inferolateral myocardium [37], which requires segmental analysis of myocardial tissue characteristics in clinical applications.

We found myocardial T1 and T2 relaxation times were longer in females than in males, which is consistent to previous 1.5T studies [9, 32]. However, there was no significant prolongation of T2 relaxation times in females in other 3T studies [9, 14, 33], which might be associated with numerous artifacts and small sample cohorts. Whether sex differences in myocardial T1 and T2 are due to biological differences or related to the different vendors and protocols remains unknown. Sex hormones are known to affect cardiac geometry and function, which were also observed in this cohort. Thinner ventricular walls in females may predispose them to partial volume effects and residual diastolic motion [38, 39]. Additionally, although HR was higher in males than in females and there was a positive correlation between HR and global T1values in males, T1 values in males are still lower than those in females, which may indicate that the influence of HR on T1 value is not dominant in this cohort.

The CMR core laboratory can provide a platform for image quality control and accurate image analysis in multicenter studies. The SD of global T1 values (34 ms) in this study was close to or smaller than previously reported single-center studies [10], ranging from 21 to 51 ms. Likewise, the SD of global T2 values (2.5 ms) was close to or smaller than reported in previous studies [26–29], ranging from about 2.3–3.3 ms. In addition, the phantom study showed strong consistency of T1 mapping and T2 mapping among centers. These results illustrate that the standardization of cardiac parametric mapping across individual sites in this study is effective and that reference values can be applied to multiple participating sites.

### Limitations

The present study has several limitations. The reference range in the present study provided only a reference for the same field strength and vendor. Contrast-enhanced imaging was not performed on healthy subjects, so we did not obtain reference ranges for post-contrast T1 and ECV, and subclinical disease or remote myocardial injury may have confounded results. The study only concerned subjects of Chinese ethnicity and did not involve direct comparisons between ethnicities. In addition, hormone levels and other serum biomarkers were not available. Correlations between native T1 and T2 and biomarkers need further exploration.

## Conclusions

In summary, using phantom-validated protocols, we systematically provide 3T reference ranges for myocardial T1 and T2 relaxation times derived from a large multicenter study of healthy Chinese adults. Myocardial T1 and T2 relaxation times were longer in females than in males. There were statistical differences in T2 across age groups, but not in T1. Our study further suggests that sex and age should be considered when interpreting myocardial T1 and T2 results. After controlling for demographic-related factors and technical heterogeneity, the present study can be referenced for the diagnosis, risk stratification, and prognostic analysis of native tissue characteristics in Chinese subjects in clinical practice and research at 3T.

### Supplementary information

**Additional file 1: Table S1.** Parameters of T1 and T2 mapping by age decades for men. **Table S2.** Parameters of T1 and T2 mapping by age decades for women.

## Data Availability

The data underlying this article will be shared on reasonable request to the corresponding author.

## Abbreviations

LV: Left ventricle/ventricular
RV: Right ventricle/ventricular
EDVi: End-diastolic volume index
ESVi: End-systolic volume index
EF: Ejection fraction
HR: Heart rate
LVMi: Left ventricular mass index
T1MES: T1 mapping and extracellular volume standardization

## References

[1] Messroghli DR, Moon JC, Ferreira VM, Grosse-Wortmann L, He T, Kellman P (2017). Clinical recommendations for cardiovascular magnetic resonance mapping of T1, T2, T2* and extracellular volume: a consensus statement by the Society for Cardiovascular Magnetic Resonance (SCMR) endorsed by the European Association for Cardiovascular Imaging (EACVI). J Cardiovasc Magn Reson.

[2] Ferreira VM, Schulz-Menger J, Holmvang G, Kramer CM, Carbone I, Sechtem U (2018). Cardiovascular magnetic resonance in nonischemic myocardial inflammation: expert recommendations. J Am Coll Cardiol..

[3] O’Brien AT, Gil KE, Varghese J, Simonetti OP, Zareba KM (2022). T2 mapping in myocardial Disease: a comprehensive review. J Cardiovasc Magn Reson.

[4] von Knobelsdorff-Brenkenhoff F, Schüler J, Dogangüzel S (2017). Detection and monitoring of acute myocarditis applying quantitative cardiovascular magnetic resonance. Circ Cardiovasc Imaging.

[5] Bohnen S, Radunski UK, Lund GK, Ojeda F, Looft Y, Senel M (2017). Tissue characterization by T1 and T2 mapping cardiovascular magnetic resonance imaging to monitor myocardial inflammation in healing Myocarditis. Eur Heart J Cardiovasc Imaging.

[6] Vermes E, Berradja N, Saab I, Genet T, Bertrand P, Pucheux J (2020). Cardiac magnetic resonance for assessment of cardiac involvement in Takotsubo syndrome: do we still need contrast administration?. Int J Cardiol.

[7] Haaf P, Garg P, Messroghli DR, Broadbent DA, Greenwood JP, Plein S (2017). Cardiac T1 mapping and extracellular volume (ECV) in clinical practice: a comprehensive review. J Cardiovasc Magn Reson.

[8] Schelbert EB, Messroghli DR (2016). State of the art: clinical applications of cardiac T1 mapping. Radiology.

[9] Granitz M, Motloch LJ, Granitz C, Meissnitzer M, Hitzl W, Hergan K (2019). Comparison of native myocardial T1 and T2 mapping at 1.5T and 3T in healthy volunteers: reference values and clinical implications. Wien Klin Wochenschr.

[10] Gottbrecht M, Kramer CM, Salerno M (2019). Native T1 and extracellular volume measurements by cardiac MRI in healthy adults: a Meta-analysis. Radiology.

[11] Hanson CA, Kamath A, Gottbrecht M, Ibrahim S, Salerno M (2020). T2 relaxation times at cardiac MRI in healthy adults: a systematic review and meta-analysis. Radiology.

[12] Liu C-Y, Liu Y-C, Wu C, Armstrong A, Volpe GJ, van der Geest RJ (2013). Evaluation of age-related interstitial myocardial fibrosis with cardiac magnetic resonance contrast-enhanced T1 mapping. J Am Coll Cardiol.

[13] Piechnik SK, Ferreira VM, Lewandowski AJ, Ntusi NA, Banerjee R, Holloway C (2013). Normal variation of magnetic resonance T1 relaxation times in the human population at 1.5 T using ShMOLLI. J Cardiovasc Magn Reson.

[14] Roy C, Slimani A, de Meester C (2017). Age and sex corrected normal reference values of T1, T2 T2* and ECV in healthy subjects at 3T CMR. J Cardiovasc Magn Reson..

[15] Dong Y, Yang D, Han Y (2018). Age and gender impact the measurement of myocardial interstitial fibrosis in a healthy adult Chinese population: a cardiac magnetic resonance study. Front Physiol..

[16] Alsaied T, Tseng SY, Siddiqui S, Patel P, Khoury PR, Crotty EJ (2021). Pediatric myocardial t1 and t2 value associations with age and heart rate at 1.5 T. Pediatr Cardiol.

[17] Schulz-Menger J, Bluemke DA, Bremerich J, Flamm SD, Fogel MA, Friedrich MG (2020). Standardized image interpretation and post-processing in cardiovascular magnetic resonance—2020 update: Society for Cardiovascular Magnetic Resonance (SCMR): Board of Trustees Task Force on Standardized Post-Processing. J Cardiovasc Magn Reson.

[18] Barral JK, Gudmundson E, Stikov N, Etezadi-Amoli M, Stoica P, Nishimura DG (2010). A robust methodology for in vivo T1 mapping. Magn Reson Med.

[19] Taylor AJ, Salerno M, Dharmakumar R, Jerosch-Herold M. T1 Mapping basic techniques and clinical applications. 2016. http://www.acc.org/jacc-journals-cme. 10.1016/j.jcmg.2015.11.005 26762877

[20] Kellman P, Hansen MS (2014). T1-mapping in the heart: accuracy and precision. J Cardiovasc Magn Reson.

[21] McDiarmid AK, Broadbent DA, Higgins DM, Swoboda PP, Kidambi A, Ripley DP (2015). The effect of changes to MOLLI scheme on T1 mapping and extra cellular volume calculation in healthy volunteers with 3 tesla cardiovascular magnetic resonance imaging. Quant Imaging Med Surg.

[22] Vassiliou VS, Wassilew K, Cameron D, Heng EL, Nyktari E, Asimakopoulos G (2018). Identification of myocardial diffuse fibrosis by 11 heartbeat MOLLI T 1 mapping: averaging to improve precision and correlation with collagen volume fraction. MAGMA.

[23] Dabir D, Child N, Kalra A, Rogers T, Gebker R, Jabbour A (2014). Reference values for healthy human myocardium using a T1 mapping methodology: results from the International T1 multicenter cardiovascular magnetic resonance study. J Cardiovasc Magn Reson.

[24] Rauhalammi SMO, Mangion K, Barrientos PH, Carrick DJA, Clerfond G, McClure J (2016). Native myocardial longitudinal (T1) relaxation time: Regional, age, and sex associations in the healthy adult heart. J Magn Reson Imaging.

[25] Rosmini S, Bulluck H, Captur G, Treibel TA, Abdel-Gadir A, Bhuva AN (2018). Myocardial native T1 and extracellular volume with healthy ageing and gender. Eur Heart J Cardiovasc Imaging.

[26] Huang L, Ran L, Zhao P, Tang D, Han R, Ai T (2019). MRI native T1 and T2 mapping of myocardial segments in hypertrophic cardiomyopathy: tissue remodeling manifested prior to structure changes. Br J Radiol.

[27] Xu Y, Sun J, Wan K, Yu L, Wang J, Li W (2020). Multiparametric cardiovascular magnetic resonance characteristics and dynamic changes in myocardial and skeletal muscles in idiopathic inflammatory cardiomyopathy. J Cardiovasc Magn Reson.

[28] Rankin AJ, Mangion K, Lees JS, Rutherford E, Gillis KA, Edy E (2021). Myocardial changes on 3T cardiovascular magnetic resonance imaging in response to haemodialysis with fluid removal. J Cardiovasc Magn Reson.

[29] Graham-Brown MPM, Gulsin GS, Poli F, Parke K, Burton JO, McCann GP (2021). Differences in native T1 and native T2 mapping between patients on hemodialysis and control subjects. Eur J Radiol.

[30] Foltz WD, Al-Kwifi O, Sussman MS, Stainsby JA, Wright GA (2003). Optimized spiral imaging for measurement of myocardial T2 relaxation. Magn Reson Med.

[31] Kawel-Boehm N, Hetzel SJ, Ambale-Venkatesh B, Captur G, Francois CJ, Jerosch-Herold M (2020). Reference ranges (“normal values”) for cardiovascular magnetic resonance (CMR) in adults and children: 2020 update. J Cardiovasc Magn Reson..

[32] Bönner F, Janzarik N, Jacoby C, Spieker M, Schnackenburg B, Range F (2015). Myocardial T2 mapping reveals age- and sex-related differences in volunteers. J Cardiovasc Magn Reson.

[33] von Knobelsdorff-Brenkenhoff F, Prothmann M, Dieringer MA, Wassmuth R, Greiser A, Schwenke C (2013). Myocardial T1 and T2 mapping at 3 T: reference values, influencing factors and implications. J Cardiovasc Magn Reson.

[34] Baeßler B, Schaarschmidt F, Stehning C, Schnackenburg B, Maintz D, Bunck AC (2015). A systematic evaluation of three different cardiac T2-mapping sequences at 1.5 and 3T in healthy volunteers. Eur J Radiol.

[35] Xu J, Zhuang B, Sirajuddin A, Li S, Huang J, Yin G (2020). MRI T1 mapping in hypertrophic cardiomyopathy: evaluation in patients without late gadolinium enhancement and hemodynamic obstruction. Radiology.

[36] Xu Z, Wang J, Cheng W, Wan K, Li W, Pu L (2023). Incremental significance of myocardial oedema for prognosis in hypertrophic cardiomyopathy. Eur Heart J Cardiovasc Imaging.

[37] Augusto JB, Nordin S, Vijapurapu R (2020). Myocardial edema, myocyte injury, and disease severity in fabry disease. Circ Cardiovasc Imaging.

[38] Wassmuth R, Prothmann M, Utz W, Dieringer M, von Knobelsdorff-Brenkenhoff F, Greiser A (2013). Variability and homogeneity of cardiovascular magnetic resonance myocardial T2-mapping in volunteers compared to patients with edema. J Cardiovasc Magn Reson.

[39] Kawel N, Nacif M, Zavodni A, Jones J, Liu S, Sibley CT (2012). T1 mapping of the myocardium: intra-individual assessment of the effect of field strength, cardiac cycle and variation by myocardial region. J Cardiovasc Magn Reson.

