# Supplementary material for: Reference ranges of myocardial T1 and T2 mapping in healthy Chinese adults: a multicenter 3T cardiovascular magnetic resonance study
Source: J Cardiovasc Magn Reson. 2023 Nov 16;25:64. doi: 10.1186/s12968-023-00974-5 (PMC10652608; doi:10.1186/s12968-023-00974-5)
Supplement: Supplementary file 1 — Additional file 1: Table S1. Parameters of T1 and T2 mapping by age decades for men. Table S2. Parameters of T1 and T2 mapping by age decades for women. [file 12968_2023_974_MOESM1_ESM.docx]

**Additional file 1**

**Table S1.** Parameters of T1 and T2 mapping by age decades for men.

| Male | 19–29 (n =145) | 30–39 (n = 106) | 40–49 (n = 88) | 50–59 (n = 110) | 60–69 (n =47) | ≥ 70 (n =19) |
| --- | --- | --- | --- | --- | --- | --- |
| T1 value (ms) | | | | | | |
| Base | 1177.3 ± 26.8  (1123.8–1230.8) | 1179.5 ± 27.8  (1123.9–1235.1) | 1183.2 ± 38.2  (1106.8–1259.6) | 1176.6 ± 34.9  (1106.7–1246.5) | 1188.9 ± 38.7  (1112.5–1267.3) | 1193.1 ± 30.6  (1132.0–1254.2) |
| Middle | 1171.8 ± 31.4  (1108.9–1234.6) | 1165.4 ± 31.3  (1102.8–1228.0) | 1166.7 ± 35.9  (1095.0–1238.5) | 1168.4 ± 33.8  (1100.9–1236.0) | 1174.5 ± 47.0  (1080.6–1268.4) | 1178.0 ± 38.6  (1100.8–1255.2) |
| Apex | 1185.9 ± 37.9  (1110.2–1261.6) | 1181.0 ± 37.0  (1107.0–1255.0) | 1178.4 ± 46.2  (1085.9–1270.8) | 1172.2 ± 44.5  (1083.2–1261.2) | 1181.1 ± 49.9  (1081.3–1280.9) | 1177.9 ± 45.6  (1086.7–1269.1) |
| Global | 1177.3 ± 25.6  (1126.1–1228.4) | 1174.3 ± 26.5  (1121.4–1227.3) | 1176.0 ± 33.5  (1109.0–1243.0) | 1172.0 ± 30.3  (111.4–1232.6) | 1182.4 ± 39.3  (1103.7–1261.1) | 1183.4 ± 29.8  (1123.8–1243.1) |
| Septum | 1189.2 ± 35.0  (1119.1–1259.2) | 1179.0 ± 36.7  (1105.5–1252.5) | 1184.2 ± 40.7  (1102.8–1265.7) | 1183.1 ± 39.7  (1103.7–1262.5) | 1194.3 ± 50.2  (1093.8–1294.8) | 1191.0 ± 47.8  (1095.4–1286.6) |
| T2 value (ms) | | | | | | |
| Base | 34.2 ± 2.6 (29.0–39.4) | 34.9 ± 2.5 (29.9–39.8) | 35.1 ± 2.5 (30.1–40.1) | 35.2 ± 2.0 (31.1–39.3) | 35.9 ± 2.3 (31.3–40.6) | 37.7 ± 2.4 (33.0–42.4) |
| Middle | 34.1 ± 2.7 (28.7–39.6) | 35.0 ± 2.6 (29.8–40.1) | 35.3 ± 2.6 (30.0–40.5) | 35.6 ± 2.4 (30.8–40.4) | 36.0 ± 2.6 (30.9–41.1) | 37.7 ± 2.7 (32.3–43.1) |
| Apex | 34.8 ± 3.1 (28.6–41.1) | 35.8 ± 3.0 (29.7–41.8) | 35.8 ± 3.1 (29.7–41.9) | 36.4 ± 2.8 (30.8–42.0) | 36.5 ± 2.6 (31.3–41.8) | 38.8 ± 2.3 (34.2–43.5) |
| Global | 34.3 ± 2.6 (29.2–39.5) | 35.1 ± 2.4 (30.3–40.0) | 35.3 ± 2.4 (30.5–40.2) | 35.6 ± 2.1 (31.4–39.9) | 36.1 ± 2.2 (31.6–40.5) | 38.0 ± 2.2 (33.5–42.4) |
| Septum | 34.7 ± 3.1 (28.6–40.9) | 35.4 ± 2.9 (29.6–41.1) | 35.6 ± 2.8 (29.9–41.3) | 36.2 ± 2.7 (30.7–41.6) | 36.7 ± 2.9 (30.8–42.5) | 38.0 ± 2.8 (32.4–43.6) |

Data are presented as means ± SD (lower/upper limits). Lower/upper limits calculated as mean ± 2 SD.

**Table S2.** Parameters of T1 and T2 mapping by age decades for women.

| Female | 19–29 (n = 130) | 30–39 (n = 86) | 40–49 (n = 98) | 50–59 (n = 129) | 60–69 (n = 45) | ≥ 70 (n = 12) |
| --- | --- | --- | --- | --- | --- | --- |
| T1 value (ms) | | | | | | |
| Base | 1207.4 ± 31.7  (1144.1–1270.8) | 1211.0 ± 29.5  (1152.0–1270.0) | 1216.3 ± 32.6  (1151.2–1281.4) | 1206.6 ± 33.7  (1139.2–1274.0) | 1222.0 ± 30.9  (1160.2–1283.9) | 1218.7 ± 32.3  (1154.0–1283.4) |
| Middle | 1202.7 ± 36.1  (1130.5–1274.8) | 1210.8 ± 32.9  (1145.1–1276.5) | 1206.6 ± 36.7  (1133.2–1280.0) | 1196.6 ± 35.3  (1126.0–1267.1) | 1211.6 ± 33.0  (1145.6–1277.6) | 1203.2 ± 28.4  (1146.4–1260.0) |
| Apex | 1225.6 ± 36.9  (1151.8–1299.5) | 1234.1 ± 32.7  (1168.8–1299.5) | 1225.1 ± 42.0  (1141.1–1309.2) | 1218.1 ± 38.3  (1141.5–1294.7) | 1223.9 ± 40.6  (1142.7–1305.1) | 1231.1 ± 40.5  (1150.0–1312.2) |
| Global | 1209.5 ± 28.6  (1152.4–1266.6) | 1215.3 ± 25.5  (1164.3–1266.4) | 1214.0 ± 31.0  (1152.0–1275.9) | 1204.2 ± 28.9  (1146.5–1261.9) | 1217.7 ± 28.7  (1160.3–1275.1) | 1215.1 ± 26.2  (1162.6–1267.5) |
| Septum | 1218.3 ± 36.8  (1144.8–1291.9) | 1222.5 ± 35.6  (1151.4–11293.7) | 1221.0 ± 38.3  (1144.4–1297.6) | 1209.3 ± 38.4  (1132.4–1286.2) | 1234.2 ± 36.5  (1161.2–1307.1) | 1227.8 ± 41.0  (1145.8–1309.8) |
| T2 value (ms) | | | | | | |
| Base | 35.8 ± 2.7 (30.3–41.2) | 36.2 ± 2.4 (31.3–41.0) | 37.1 ± 2.0 (33.1–41.1) | 36.4 ± 2.5 (31.4–41.3) | 37.0 ± 2.5 (32.1–42.0) | 37.6 ± 2.4 (32.8–42.4) |
| Middle | 36.2 ± 2.6 (31.0–41.4) | 36.4 ± 2.6 (31.2–41.6) | 37.1± 1.9 (33.2–40.9) | 36.6 ± 2.7 (31.2–41.9) | 37.3 ± 2.6 (32.0–42.6) | 38.3 ± 2.6 (33.2–43.5) |
| Apex | 36.6 ± 3.0 (30.6–42.7) | 36.8 ± 3.0 (30.9–42.7) | 37.9 ± 2.5 (33.0–42.9) | 37.2 ± 2.9 (31.3–43.0) | 37.6 ± 3.0 (31.5–43.6) | 39.2 ± 1.7 (35.8–42.6) |
| Global | 36.2 ± 2.5 (31.2–41.1) | 36.4 ± 2.4 (31.6–41.2) | 37.3 ± 1.8 (33.7–40.8) | 36.6 ± 2.4 (31.9–41.4) | 37.2 ± 2.5 (32.2–42.2) | 38.2 ± 1.9 (34.5–42.0) |
| Septum | 36.7 ± 3.0 (30.8–42.7) | 36.8 ± 2.9 (30.9–42.7) | 37.5 ± 2.1 (33.3–41.8) | 36.6 ± 3.1 (30.4–42.9) | 37.3 ± 2.6 (32.0–42.6) | 38.7 ± 2.8 (33.1–44.3) |

Data are presented as means ± SD (lower/upper limits). Lower/upper limits calculated as mean ± 2 SD.
